# Hypidone Hydrochloride (YL-0919) Produces a Fast-Onset Reversal of the Behavioral and Synaptic Deficits Caused by Chronic Stress Exposure

**DOI:** 10.3389/fncel.2018.00395

**Published:** 2018-11-20

**Authors:** Yuhua Ran, Zengliang Jin, Xiaofei Chen, Nan Zhao, Xinxin Fang, Liming Zhang, Youzhi Zhang, Yunfeng Li

**Affiliations:** ^1^State Key Laboratory of Toxicology Medical Countermeasures, Beijing Key Laboratories of Neuropsychopharmacology, Beijing Institute of Pharmacology and Toxicology, Academy of Military Sciences, Beijing, China; ^2^Department of Pharmacology, School of Basic Medical Sciences, Capital Medical University, Beijing, China

**Keywords:** YL-0919, fast-onset, mTOR, anti-depressant, chronic unpredictable stress

## Abstract

Our previous study showed that hypidone hydrochloride (YL-0919), a partial serotonin 1A (5-HT_1A_) receptor agonist and 5-HT reuptake inhibitor, exerts a significant antidepressant effect in various animal models. The aim of the present study was to further investigate the underlying mechanisms and whether it could act as a fast-onset antidepressant. In the current study, depressive-like behavior was induced in rats by a chronic unpredictable stress (CUS) model and assessed with the Sucrose Preference Test (SPT). Treatment with YL-0919 (2.5 mg/kg, i.g.), but not with fluoxetine (Flx; 10 mg/kg, i.g.), caused a fast improvement in the SPT scores. In CUS-exposed rats, YL-0919 treatment for 5 days decreased the immobility time in a forced swimming test (FST), and a 10-day treatment decreased the latency to feed in a Novelty-Suppressed Feeding Test (NSFT). In addition to the behavioral tests, the effects of YL-0919 on synaptic protein expression were also evaluated. Western blotting showed that YL-0919 significantly enhanced the expression levels of synaptic proteins such as synapsin I, postsynaptic density protein 95 (PSD95), phosphorylated mammalian targeting of rapamycin (pmTOR) and brain-derived neurotrophic factor (BDNF) in the hippocampus. To determine how the mTOR signaling is involved in the fast-onset antidepressant-like effects of YL-0919, the mTOR-specific inhibitor rapamycin was administered intracerebroventricularly (i.c.v.) together with the YL-0919 treatment. The observed changes in behavioral tests and protein expression could be reversed by rapamycin treatment. This suggests that the fast-onset antidepressant effects of YL-0919 were partially caused by changes in synaptogenesis mediated by activation of mTOR pathways. Our data suggest that YL-0919 may be a powerful/effective antidepressant with fast-onset.

## Introduction

Major depressive disorder is a serious neuropsychiatric condition that affects a large number of people worldwide (Kessler et al., 2003). The antidepressants currently available in clinical settings mainly target noradrenaline/serotonin (NE/5-HT) transporters; however, these agents usually exhibit a time lag of weeks in their effects and show initial response rates of only around 33%. Chronic treatments must be a long-lasting course and the antidepressant treatment commonly needs several weeks to months to create an effect (Penn and Tracy, 2012). Novel agents that can induce the fast onset of robust antidepressant responses are needed to alleviate the challenges of the treatment of depression.

As an NMDA receptor antagonist, a single intravenous administration of ketamine, induced a rapid and long-lasting antidepressant effect within 2 h, that lasted almost 1 week in clinical trials (Berman et al., 2000; Zarate et al., 2006). However, the side-effects of addiction and the potential of drug abuse associated with ketamine, have severely restricted its clinical use (Liston et al., 2006; Liebrenz et al., 2007).

Therefore, efforts are now focused on developing fast-onset and effective medication that can induce prompt antidepressant responses with minor side effects or abuse liability. Ketamine reverses the loss of excitatory spine synapses in the medial prefrontal cortex (PFC), within 24 h after exposure to chronic unpredictable stress (CUS; Zarate et al., 2006). These ketamine-induced effects on the behavioral and neuronal deficits induced by CUS require regulation via the mammalian target of rapamycin complex 1 (mTORC1) signaling (Yilmaz et al., 2002; Liebrenz et al., 2007). mTOR activation has been functionally connected to local protein synthesis in synapses such as postsynaptic density protein 95 (PSD95) and synapsin I (Hoeffer and Klann, 2010). Recent research suggests the mTOR signaling pathway could be a potential fast-onset target of antidepressant drugs.

An imbalance in 5-HT levels, 5-HT receptors coding gene polymorphism and 5-HT transporter can influence brain functions in such a way that it leads to depression. 5-HT, as a neurotransmitter, extensively participates in the pathophysiological processes of depression. Conventional antidepressants show their effectiveness by increasing the level of monoamines, which share mechanisms of action identical to tramadol (Yang et al., 2012). Pretreatment with tramadol, a widely used analgesic agent, enhanced the ketamine-induced antidepressant effects and upregulated the expression of mTOR in rat hippocampus and PFC (Yang et al., 2012). It exerts therapeutic effects via activation of opioid receptors and elevation of the plasma levels of 5-HT and norepinephrine (Barber, 2011). Hoeffer and Klann (2010) showed that phosphorylated and activated forms of extracellular signal-regulated kinase (ERK/PKB/AKT) signaling pathways, linked to the activation of mTOR signaling, are involved in the anti-depressive effects of ketamine. Imipramine could cause antidepressive effects via PI3K/Akt/mTOR signaling (Jeon et al., 2011). By analyzing, we hypothesize that antidepressants initiate the 5-HT-mediated brain-derived neurotrophic factor (BDNF) biosynthesis through inhibiting the 5-HT reuptake transporter, then promoting the BDNF downstream signaling cascades (MAPK/ERK and/or PI3K/AKT pathways), and finally inducing the mTOR phosphorylation causing antidepresssive effects. Current studies show that ketamine activates mTOR through AMPA receptors, which seems to have a relatively shorter (a few minutes) pathway compared to other antidepressants (Xu et al., 2018). Pharmacological agents that target serotonergic receptors have been reported to induce fast-onset antidepressant effects in rodents, including the 5-HT_2C_ receptor antagonist (Opal et al., 2014), 5-HT_4_ agonists (Lucas et al., 2007) and 5-HT_7_ antagonists (Mnie-Filali et al., 2011).

Hypidone hydrochloride (YL-0919) has dual 5-HT_1A_ effects as a partial receptor agonist and a selective reuptake inhibitor (Chen et al., 2013). Previous studies show that YL-0919 exerts reliable antidepressant and anxiolytic-like effects and does not result in a marked inhibition of sexual functions (Baldwin and Foong, 2013). Data from another study also demonstrate that YL-0919, together with its significant antidepressant- and anxiolytic-effects, induces a greater impact on extracellular 5-HT levels than the conventional selective 5-HT reuptake inhibitor fluoxetine (Flx). Furthermore, treatment with YL-0919 (7 days vs. Flx 21 days) affected hippocampal synaptic plasticity, thus enhancing long-term potentiation (LTP) in rats, faster than Flx (Zhang et al., 2017).

These data suggest that YL-0919 is a fast-onset and powerful antidepressant with fewer side effects than classic a SSRI antidepressant such as Flx (Ran et al., 2017), which led to further investigations of its underlying mechanisms. Based on the findings described above, YL-0919 seems to be a novel type of antidepressant with the potential to yield fast-onset effects. Moreover, it has many advantageous pharmacological properties, such as fewer side effects and good physiochemical qualities. Until now, it is not clear whether the YL-0919-mediated induction of mTOR induces similar fast-onset effects. These data suggest that YL-0919 is a novel type of antidepressant with many advantageous pharmacological properties such as a fast onset, high efficacy, good physiochemical qualities and fewer side effects. This promising new antidepressant class led to further investigations of its underlying mechanisms.

## Materials and Methods

### Animals

ICR mice (male, 18 ± 2 g) and Sprague-Dawley rats (male, 180 ± 10 g) were purchased from the Beijing Vital Laboratory Animal Technology Company (Beijing, China). The animals were housed under standard environmental conditions with controlled a humidity (45%), temperature (23 ± 1°C), and lighting (12 h/day). The experiments were carried out according to the National Institute of Health Guide for the Care and Use of Laboratory Animals (NIH Publications No. 80-23, revised in 1996). All procedures were approved by the Institutional Committee on Animal Care and Use (IACUC) of the Institute of Pharmacology and Toxicology. All efforts were made to minimize animal suffering and reduce the number of animals used for the experiments.

### Drugs and Reagents

Rapamycin, Flx and DMSO purchased from Sigma-Aldrich (St. Louis, MO, USA). YL-0919 (solid powder, purity ≥99% tested by high performance liquid chromatography) was synthesized by the Department of Medicinal Chemistry at our institute. Unless otherwise specified, YL-0919 (2.5 mg/kg) and Flx (10 mg/kg) were dissolved in saline and administered i.g. Rapamycin (0.2 nmol) was dissolved in DMSO and administered to mice or rats via an intracerebroventricular (i.c.v.) injection and the dosage for the specific target was chosen based on previous report (Cota et al., 2006).

### Drug Administration and Surgical Procedures

#### Acute Experiments

**Figure 1:**
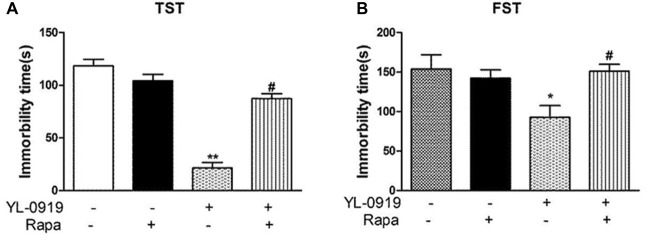
Effects of YL-0919 and rapamycin (Rapa) on depression-like behavior in mice. **(A)** Tail Suspension Test (TST) and **(B)** Forced Swimming Test (FST). Rapamycin was administered intracerebroventricularly (i.c.v.) at a final concentration of 0.2 mmol in 2 μL DMSO, with DMSO as a vehicle control 12 h before treatment with YL-0919 (2.5 mg/kg) or fluoxetine (Flx; 10 mg/kg, both-administered i.g.). The TST and FST were performed 1 h after YL-0919 application. *n* = 8; *^,#^*P* < 0.05, **P < 0.01; *^,#^compared to vehicle and YL-0919, respectively; two-way ANOVAs followed Tukey’s *post hoc* test.

In acute experiments, mice received a single i.c.v. administration of the specific mTOR inhibitor rapamycin (0.2 nmol in 2 μL DMSO) 12 h before the administration of YL-0919. Behavioral tests such as the Tail Suspension Test (TST) and Forced Swimming Test (FST) were carried out 1 h after YL-0919 or Flx application (see these results in Figure 1).

#### Chronic Experiments and Surgical Procedure

**Figure 2:**
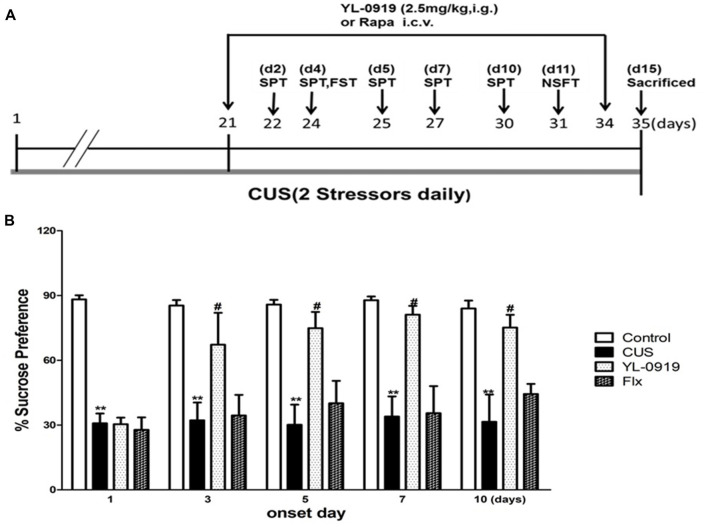
**(A)** YL-0919 induced rapid anti-depressant responses in a chronic unpredictable stress (CUS) paradigm. Schematic drawing demonstrating the timeline for CUS exposure, drug administration and behavioral testing. Numbers in parentheses represent days after drug administration. Rats were exposed to CUS for 35 days and administered YL-0919 (2.5 mg/kg, i.g.) or Flx (10 mg/kg, i.g.) from day 21 onwards and lasting for 14 days. **(B)** Effects of YL-0919 on CUS-induced depressive-like behavior in rats assessed by Sucrose Preference Test (SPT) on day 1, 3, 5, 7 and 10 after drug administration. *n* = 8; **^,#^*P* < 0.05 compared to vehicle and YL-0919, respectively; two-way ANOVAs followed Tukey’s *post hoc* test.

Rats received an DMSO vehicle or rapamycin (0.2 nmol in 2 μL DMSO) i.c.v. once per day according to their group, starting on day 1 of the CUS treatment and lasting for 35 days. Based on previous studies (Li et al., 2010), 1 h after the rapamycin treatment, YL-0919 or Flx was intragastrically administered. The animals were assessed based on behavioral tests using the following paradigms, and tissues were collected for western blotting assays on day 35 as described below (see Figure 2A).

For experiments involving the central administration of rapamycin, guide cannulae (22G) were implanted into the lateral ventricles of rats (coordinates from Bregma: −0.9 mm anterior/posterior, −1.5 mm medial/lateral and −3.3 mm dorsal/ventral from dura) before the start of the CUS (Banasr et al., 2007; Li et al., 2011). The surgical procedures were carried out under chloral hydrate (Damao, Tianjin, China) intraperitoneal (i.p.) injection at a dosage of 8 mg/kg. During recovery, animals carried a dummy cannula. After 1-week of recovery, CUS was induced according to the paradigms mentioned above, for 35 days. On day 21 of the CUS treatment, a Sucrose Preference Test (SPT) was performed to verify the success of these stresses treatment, as the SPT scores remarkably decreased in CUS-treated rats compared to normal controls (data not shown). From day 1 to 35, a rapamycin or DSMO vehicle was delivered daily at a rate of 0.25 μL/min with an injection cannula (26G) protruding 0.5 mm beyond the guide cannula (RWD Technology, Shenzhen). After 30 min, the animals additionally received YL-0919. The injection cannula remained in the guide cannula for at least 1 min after each infusion. Depending on the group, the rats additionally received either YL-0919 or Flx.

### CUS Procedure in Rats

Our group has successfully established a CUS paradigm and verified the antidepressant effects of YL-0919 in this model (Banasr et al., 2007; Banasr and Duman, 2008; Ran et al., 2017). Animals were exposed to a variable sequence of mild and unpredictable stressors for 21 days. The procedure consisted of exposure to a sequence of 10 stressors (two stressors per day), which included food or water deprivation, aversive odor, no lighting for 3 h (10:00 AM–1:00 PM), lighting overnight, strobe lights overnight, changes in ambient temperature upto 4°C, crowded and isolated housing, rotation on a shaker and 45° tilted cages. It was verified with a SPT test that these procedures induced depressive-like behavioral changes.

### Behavioral Tests

#### Forced Swimming Test (FST) in Mice

The FST was performed in mice according to Porsolt’s protocol (Porsolt et al., 1978; Zhang et al., 2014) with minor modifications. The mice were randomly grouped and received a rapamycin administration and a single YL-0919 or Flx treatment. One hour after intragastric administration of the compounds (i.g.), the mice were placed individually in cylindrical containers (diameter, 12 cm; height, 20 cm; containing 10 cm of water maintained at 25°C). The immobility time in the last 4 min of a total of 6 min was recorded. Floating motionlessly or making only movements necessary to keep the head above the water were considered immobility time.

#### Forced Swimming Test in Rats

The FST in rats used the same procedure as the test for mice described earlier in “Forced Swimming Test (FST) in Mice” section (Porsolt et al., 1978; Zhang et al., 2014). The procedure included two parts, a pre-test and test session, which were performed using the same apparatus and conditions (diameter, 20 cm; height, 40 cm; containing 25 cm of water maintained at 25°C). During the pre-test session, rats were forced to swim for 15 min; 24 h later, the rats were placed in the same apparatus for 5 min, and this was designated as the test session. The total time of immobility (defined as described in “Forced Swimming Test (FST) in Mice” section) during this 5 min period was measured.

#### Tail Suspension Test (TST) in Mice

The TST was performed in mice as reported previously (Steru et al., 1985) with minor improvements. Mice were suspended from the top of an apparatus using adhesive tape that was placed approximately 1 cm from the tip of the tail. The total immobility time in the last 4 min of a total of 6 min was recorded. Mice were classified as immobile when they hung passively without moving.

#### Sucrose Preference Test (SPT) in CUS Rats

The SPT procedure was adapted from previous studies (Cryan and Leonard, 2000; Ran et al., 2017). In summary, rats were given a palatable 1% sucrose water solution (Sigma-Aldrich) for 48 h for acclimatization. They were then exposed to water deprivation for 4 h, followed by a 1 h exposure to two identical bottles, one filled with sucrose solution and the other with water. Sucrose and water consumption were determined by measuring the bottle weight before and after the test, so as to calculate the sucrose preference rate. A water test was performed in a similar manner on days 1 (22 days CUS), 3 (24 days), 5 (26 days), 7 (28 days) and 10 (31 days) after the first YL-0919 or Flx administration (Figure 2A).

#### Novelty-Suppressed Feeding Test (NSFT) in CUS Rats

The Novelty-Suppressed Feeding Test (NSFT) was performed as described previously (Santarelli et al., 2003). Before testing, rats were deprived of food overnight. Rats were individually assessed in an open, white, plastic chamber (76.5 × 76.5 × 40 cm). The floor of the chamber was covered by a 1-cm thick layer of wooden bedding, and four pellets of food (regular chow) were placed in the center. The latency to feed, specifically the time it took the animal to approach and take the first bite of the food, was recorded for up to 5 min. As a control value, food intake in the home cage was measured immediately after the test, post the 10-day treatment with YL-0919.

### Western Blotting

Hippocampi of rats from different groups were dissected on the 35th CUS day. The hippocampi were rapidly frozen and lysed in buffer containing protease and phosphatase inhibitors (Li et al., 2011). Total protein concentration was quantified by Bradford analysis. Protein quantification was carried out by sodium dodecyl sulfate-polyacrylamide gel electrophoresis. Primary antibodies (Cell Signaling Technology, Beverly, MA, USA) against β-actin and BDNF were diluted 1:10,000 and 1:2,000, respectively. The following additional primary antibodies were used at a dilution of 1:500—synapsin I, PSD95, phosphorylated mTOR (pmTOR) and total mTOR. Anti-rabbit secondary antibodies were used at a dilution of 1:10,000. Bands developed using enzymatic chemiluminescence were exposed to films, which were analyzed on a Gel-Pro Analyzer (Media Cybernetics, Bethesda, MA, USA). BDNF pmTOR, total mTOR, synapsin I and PSD95 were normalized to β-actin.

### Statistical Analysis

All data are described as the mean ± standard error. Two-way ANOVAs were used to analyze the difference between experimental groups in SPT, FST, TST, NSFT and protein expression. The first element included the Control/CUS and Vehicle/YL-0919 groups while the second included the Vehicle/YL-0919 and Rapamycin/Rapamycin + YL-0919 groups from the four CUS groups. These were then followed by the Tukey’s *post hoc* test to verify that CUS can induce various effects that can be reversed by YL-0919, a treatment that itself is reversed by rapamycin. A *P* value less than 0.05 was considered as significant. The significance is labeled in the figures as ^#,^*^,$^*P* < 0.05 and **P < 0.01.

## Results

### YL-0919 Rapidly Reversed Acute Behavioral Deficits in Mice

As shown in Figures 1A,B, acute treatment with YL-0919 (2.5 mg/kg, p.o.) significantly decreased the immobility time of mice in the TST (*P* < 0.01 vs. vehicle). YL-0919 also markedly reduced the immobility time in the FST (*P* < 0.05 vs. vehicle). In another set of experiments, the YL-0919-induced decrease in the immobility time, was reversed by the mTOR inhibitor rapamycin (0.2 nmol i.c.v.; *P* < 0.05 vs. YL-0919-treated mice) in both the TST and FST, but rapamycin treatment alone did not alter the immobility time.

### YL-0919 Rapidly Reversed the CUS-Induced Behavioral Deficits in Rats

**Figure 3:**
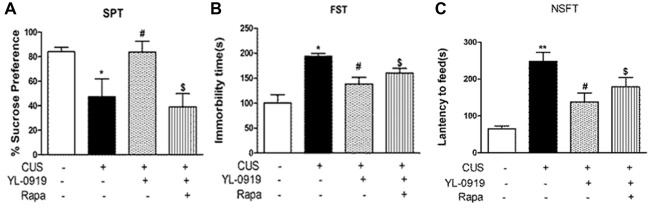
Effects of rapamycin (0.2 nmol in 2 μL DMSO, i.c.v.) on behavioral tests assessing depressive-like behavior in rats exposed to CUS. YL-0919 (2.5 mg/kg, i.g.) or saline were administered i.g. 1 h after rapamycin treatment. **(A)** Effects of rapamycin on the SPT score of CUS-exposed rats after 3 days treatment with YL-0919. **(B)** Effects of rapamycin on the FST score of CUS-induced rats after treatment with YL-0919 for 5 days. **(C)** Rapamycin (Rapa) effects on the novelty-Suppressed Feeding Test (NSFT) in rats exposed to CUS after 10 days treatment with YL-0919. All behavioral tests were completed 1 h after drug administration. *n* = 8; *^,#^*P* < 0.05, **P < 0.01; *^,#^compared to vehicle and YL0919, respectively; ^$^*P* < 0.05, compared to Rapa; two-way ANOVAs followed Tukey’s *post hoc* test.

Rats exposed to CUS, which is a classical and commonly used model of depression, develop depressive behaviors, such as anhedonia and reversal of these effects requires chronic antidepression treatment (Willner, 2005). In the present study, CUS-exposed rats exhibited a reduction in the SPT rate compared to control rats, which is an indicator of depressive behavior (Figure 2B). The SPT performance of both CUS and control animals remained consistent for up to 10 days, indicating that the animals did not habituate after repeated testing. Additionally, the rats exposed to CUS demonstrated an increased latency to feed in the NSFT (Figure 3C). Daily administration of YL-0919 increased the sucrose preference rate after 3 days (Figure 3A). To confirm this YL-0919 effect, the sucrose preference rate was also determined at days 5, 7 and 10 after the first YL-0919 administration. Compared to control rats, YL-0919 treatment caused a consistent and significant increase in the sucrose preference rate (Figure 2B).

### YL-0919-Induced Reversal of the Behavioral Deficits Caused by CUS Involved mTOR Signaling

YL-0919 showed a powerful effect in the SPT from the third day onward after drug treatment, which implies that it has sustained effects (Figure 3). In contrast, the time required for Flx (10 mg/kg, p.o.) to start exhibiting its antidepressant effect was more than 10 d after the first drug administration. Previous studies have reported that YL-0919 exhibits antidepressant actions in a series of behavior tests (Chen et al., 2013; Ran et al., 2017; Zhang et al., 2017). Here, we found that the fast-onset effect of YL-0919 is related to mTOR signaling as determined by behavioral tests such as SPT, NSFT, TST and FST. In our study, we have shown that the administration of rapamycin leads to the reversal of the YL-0919 effect on the behavioral deficits caused by CUS exposure in both the SPT and NSFT, 10 days after drug administration (Figures 3A,C). The same effects of rapamycin on YL-0919 were shown in the FST after 10 days of treatment with YL-0919. It can be inferred that continuous treatment with YL-0919 had an antidepressant effect that could be reversed by rapamycin.

Rapamycin reversed the antidepressant-like effects of YL-0919 in the TST and FST (Figures 1A,B). As described earlier, YL-0919 completely reversed the effect of CUS-induced behavioral deficits in the SPT and NSFT in rats (Figure 3). These effects of YL-0919 were completely blocked by rapamycin treatment (Figure 3). Rapamycin given for 35 days, in the absence of YL-0919 and in stressed rats, had no effect in either test.

### YL-0919 Rapidly Reverses CUS-Induced Deficits in Synaptic Proteins Through mTOR Signaling

**Figure 4:**
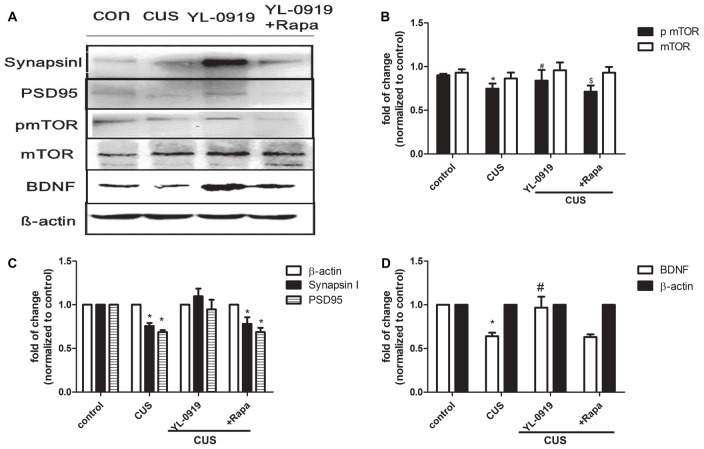
Hippocampal proteins expression of synapsin I, postsynaptic density protein 95 (PSD95), phosphorylated mammalian targeting of rapamycin (pmTOR), mTOR and and brain-derived neurotrophic factor (BDNF) in CUS-induced depressive-like rats treated with YL-0919 and/or rapamycin for 14 days. Rats were exposed to CUS and infused on day 21 with vehicle or rapamycin (0.2 nmol in DMSO, i.c.v.) 1 h before YL-0919 (2.5 mg/kg, i.g.) or vehicle treatment for 14 days. **(A)** The representative images of proteins levels of synapsin I, PSD95, pmTOR, mTOR and BDNF were determined by western blots are shown. **(B)** Levels of synapsin I and PSD95 were quantified with β-actin. **(C)** Levels of pmTOR were quantified with each β-actin and mTOR were quantified with each β-actin respectively, then the ratio of pmTOR/mTOR were as below. **(D)** Levels of BDNF were quantified with β-actin. Results were quantified and are the mean ± SEM, percent of control (*n* = 4 animals; *^,#,$^*P* < 0.05, two way ANOVAs followed Tukey’s *post hoc* test).

Chronic stress paradigms have been demonstrated to profoundly alter brain structures and functions in rodents, causing the atrophy of pyramidal neurons in the PFC and hippocampus (Joels et al., 2004; Radley and Morrison, 2005; Liston et al., 2006; Radley et al., 2006, 2008; Liu and Aghajanian, 2008). We conducted experiments to determine whether YL-0919 alters the expression of synaptic proteins and whether rapamycin can reverse these effects. Rapamycin application decreased the expression of pmTOR in CUS rats treated with YL-0919 (Figure 4C). CUS treatment for 35 days, markedly decreased the levels of several well-characterized synaptic proteins in synaptoneurosome preparations of the hippocampus (Figure 4B), including the presynaptic protein synapsin I and the postsynaptic protein PSD95. In contrast, daily administration of YL-0919 for 10 days, combined with daily CUS exposure completely reversed these deficits in synaptic proteins (Figure 4). The ability of YL-0919 to reverse the deficits in synapsin I and PSD95 was blocked by i.c.v. infusion of rapamycin (Figure 4B). Further analysis in another study showed that treatment with YL-0919 for 10 days continued to reverse the CUS-induced deficits in the expression of mTOR signaling (Figure 3).

## Discussion

The data obtained in the current study demonstrate that YL-0919, which is a partial 5-HT_1A_ receptor agonist and a selective serotonin reuptake inhibitor (SSRI), induces an antidepressant effect in rats after 3 days of treatment, which is significantly faster than conventional SSRIs such as Flx, and significantly influences synaptogenesis as observed by the expression of synaptic proteins such as synapsin I and PSD95. These antidepressant effects of YL-0919 treatment could be reversed by the mTOR-specific inhibitor rapamycin, which could also block the upregulation of synaptic proteins caused by YL-0919. These data suggest that the fast-onset antidepressant-like effects of YL-0919 could occur via regulation of the synaptogenesis in the PFC and involve mTOR signaling.

To reach a reliable conclusion, both acute and chronic models of depression were used in our study, as they are widely acknowledged models which mimic many depressive-like symptoms (Qiao et al., 2016). We first reproduced the antidepressant effects of YL-0919 in the two types of models of depression, providing the effectiveness of YL-0919. Moreover, acute models including those of FST and TST were introduced to verify the effect of rapamycin on depressive-like behavior. Our studies demonstrate that the specific inhibitor of rapamycin alone has no influence on depressive-like mice. The data also shows that the antidepressant effect of YL-0919 could be reversed by co-administration of rapamycin. It suggests that mTOR signaling plays a role in the effect of YL-019. CUS models were then applied to investigate the possible fast-onset action of YL-00919 and the interaction with mTOR signaling.

In this study, we first examined the acute influence of rapamycin on YL-0919 effects in the TST and FST in rats, and found that i.c.v. application of rapamycin alone did not influence their behavior. Rapamycin did, however, block the antidepressant effects of YL-0919. This suggests that mTOR could be implicated in the antidepressant effects of YL-0919. Generally, it was thought that the pharmacological inhibition of mTOR by rapamycin would have a wide range of clinical effects. Rapamycin-based therapy has shown benefits for patients with renal cancer carcinoma, tuberous sclerosis complex, lymphangioleiomyomatosis-related tumors, and metabolic diseases such as obesity and diabetes, even in longevity and aging. However, the use of rapamycin monotherapy in a broad spectrum of metabolic diseases and cancers, is limited due to its modest efficacy. At present, there are no reports on the direct use of rapamycin monotherapy or combination therapy, for depression. Rapamycin alone could neither induce nor aggravate depressive-like behavior, possibly because its limited existing bio-actives may not directly or indirectly reduce mTORC1 and/or mTORC2 activity, which are involved with several feedback loops and signaling pathways affecting the underlying mechanisms of depression (Abelaira et al., 2014). Next, the fast-onset effects of YL-0919 were evaluated in rats with CUS-induced depression using the SPT (Goldwater et al., 2009). The CUS model allows the detection of rapidly occurring drug effects. Typical antidepressants fail to reverse the effects of chronic stress even after 1 week of treatment (Papp et al., 1996). We found that YL-0919 was able to increase the sucrose preference rate after only 3 days of treatment, while the first-line SSRI Flx did not show this rapid action. Even 10 days of treatment with Flx could not increase the sucrose preference rate significantly. This is in accordance with studies reporting a time lag of 2–4 weeks before currently available medications exert an antidepressant effect (Penn and Tracy, 2012).

It is thought that an indirect reduction in serotonergic transmission via the activation of somatodendritic 5-HT_1A_ receptors in the raphe nuclei, with resulting reduction of 5-HT release, plays an important role in the delayed onset of therapeutic SSRI actions. It has been suggested that 5-HT partial agonist and reuptake inhibitor may theoretically speed up the onset of antidepressant-like activity (Hogg and Dalvi, 2004). In contrast to acute treatment, chronic treatment with antidepressants reduces hyponeophagia (Sartori et al., 2012; Duman and Newton, 2013). The SPT is a sensitive and reliable method to study the time course of antidepressant efficacy (Li et al., 2010). In our study, the sucrose preference rate was significantly increased after 3 days, 7 days, 10 days and 14 days of treatment with YL-0919, while chronic treatment for 10 days with Flx did not affect this parameter. This result is in agreement with previous data from our group that subchronic (7 days) and chronic (21 days) treatment with YL-0919 both lead to reduced feeding latency, whereas subchronic administration of Flx does not affect behavior in the NSFT (Zhang et al., 2017). Golgi staining data showed that YL-0919 enhances the length and numbers of dendrites of CUS-induced rats after chronic treatment (Ran et al., 2017).

It has been reported that antidepressants increase the expression of synaptic proteins and lead to behavioral changes. This effect could be blocked by rapamycin (Li et al., 2010). Thus, we determined the expression of synaptic proteins such as synapsin I and PSD95. The data demonstrate that rapamycin blocks the effects of YL-0919 on behavioral deficits caused by CUS and indicate that the effects of YL-0919 involve mTOR signaling to alter dendrite spine or synapse composition. This is consistent with previous reports on mTOR activity (Ornstein et al., 1998; Baldwin and Foong, 2013). In support of this hypothesis, we found that CUS exposure decreases synapsin I and PSD95 levels in the hippocampus; an effect that was rapidly reversed by YL-0919 in a rapamycin-dependent manner. We also examined the possibility that mTOR inhibition underlies the decrease in synaptic proteins caused by CUS after 15 days of treatment with YL-0919 and found that there were significant differences in levels of phosphorylated/activated mTOR and BDNF (Figure 4). We found that CUS induced decreases in BDNF levels, which is the same phenomenon observed in other CUS-induced rats (Ran et al., 2017). YL-0919 could reverse the decrease in BDNF levels, and rapamycin was able to block this effect (Figure 4). In this study, although we could not determine the mTOR-related protein expression treated by Flx, but there is some evidence in to support it. Liu et al. (2015) found that chronic Flx treatment attenuated the CUMS-induced pmTOR reduction in the hippocampus and amygdala of mice but not in the frontal cortex or the hypothalamus. Moreover, the CUMS-decreased PSD-95 and synapsin I levels were reversed by Flx, and these effects were blocked by rapamycin only in the hippocampus (Liu et al., 2015). The decrease in synaptic proteins could also be mediated by other potential mechanisms, such as enhanced degradation or other intracellular molecules upstream of the mTOR signaling pathways (Gourley et al., 2008; Maeng et al., 2008; Li et al., 2011). The activation of CREB increases BDNF transcription and expression, required for the antidepressant behavioral response to current antidepressants (Duman and Voleti, 2012). Our present findings suggest that BDNF induction remains the molecular marker that correlates most consistently with antidepressant onset (Duman, 2004). Our findings also indicate substantial overlap of the molecular signaling pathways mediating fast-onset ketamine. These findings show that agents acting on 5-HT-like YL-0919 are capable of inducing fast-onset antidepressant effects.

In Figure 4, normalization β-actin is recognized as an internal reference. For most tissues and cells, its expression is abundant and stable. In response to this issue, I think it is necessary to explore the rationality of β-actin as an internal reference. First of all, the actin family includes 6 proteins. The homology between different actins is as high as 90%. There are four muscle tissue-specific β-actin, and the other two are expressed in non-muscle tissues. The sample in this study is brain tissue, not muscle tissue. So specificity is not a problem. According to the following literature reports, there are three cases of β-actin changes. (1) In mouse spinal cord injury or muscle atrophy models, as well as some neurodegenerative diseases, the expression of actin will change (Ferguson et al., 2005; Bauer et al., 2009); (2) Changes in expression levels in different tissues of the same species. Studies have analyzed that the expression levels of actin in muscle, heart, bone, and fat of mice are different (Eaton et al., 2013); (3) Changes in the expression of actin in different parts of the same tissue. In the sciatic nerve tissue of mice, the expression of actin is higher in the proximal tissue than in the distal tissue. Same as above mentioned, effective linear range of β-actin. An ideal internal control should have a wide linear interval to accommodate samples with different protein expression levels. Eaton et al.'s thermal study found that in brain homogenate, the linearity of β-actin was better when the total protein loading amount was between 10–30 μg, and the band signal above 30 μg tended to be saturated. However, the experiment (Dittmer and Dittmer, 2006) found that 2 μg was saturated. This difference may be related to the experimental sample and the sensitivity of fluorescence chromatography. The specific brain samples were collected to investigate the proteins expressions with β-actin as internal control is reasonable and admitted. In fact, there is none even possible minor element to have effect on the expression of β-actin in this study. Of course, we know that the sensitivity of fluorescence chromatography is much higher than that of ECL luminescence method. Based on the above research on β-actin as internal reference combined with the western blot results in Figure 4 of our article, we unanimously agree and recognize that the quantitative method of western blot mentioned by experts is the most correct and accurate, but in our experiment, it did not involve factors that cause actin to change. Any factor, therefore, we adopted an approach also adopted by many other colleagues to compare target proteins. This is of course not as accurate as the quantitative method mentioned by experts, but our method simplifies the steps. Although the process is not precise, it does not affect the final result. Biomedicine is developing rapidly, and western blot, as a protein semi-quantitative technology, is also undergoing innovation.

In summary, our results provide direct evidence that YL-0919 rapidly reverses the behavioral, physiological and morphological deficits induced by chronic stress. These effects are mediated via a rapamycin-sensitive mTOR pathway. These findings, together with earlier studies, provide evidence that YL-0919 is a safer antidepressant medication with a faster onset and fewer side effects. To verify these findings, some other methods such as histochemistry and photo-genetics technology should be also be tested in the future. Studies are underway to test additional targets in the BDNF-mTOR signaling pathway that could potentially lead to other rapid-acting antidepressants.

## Author Contributions

YR performed the research design, behavior test, data analysis and wrote the manuscript. ZJ and XC performed the molecular chemistry tests. NZ, XF and LZ supported the completion of the animal behavior tests. YZ and YL contributed to the research design and revised the manuscript.

## Conflict of Interest Statement

The authors declare that the research was conducted in the absence of any commercial or financial relationships that could be construed as a potential conflict of interest.
