# Corrigendum: Hypidone hydrochloride (YL-0919) produces a fast-onset reversal of the behavioral and synaptic deficits caused by chronic stress exposure

**DOI:** 10.3389/fncel.2025.1558690

**Published:** 2025-03-31

**Authors:** Yuhua Ran, Zengliang Jin, Xiaofei Chen, Nan Zhao, Xinxin Fang, Liming Zhang, Youzhi Zhang, Yunfeng Li

**Affiliations:** ^1^State Key Laboratory of Toxicology Medical Countermeasures, Beijing Key Laboratories of Neuropsychopharmacology, Beijing Institute of Pharmacology and Toxicology, Academy of Military Sciences, Beijing, China; ^2^Department of Pharmacology, School of Basic Medical Sciences, Capital Medical University, Beijing, China

**Keywords:** YL-0919, fast-onset, mTOR, anti-depressant, chronic unpredictable stress

In the published article, there was an error in the legend for **Figure 4C** as published. The corrected legend for **Figure 4C** appears below:

“**(C)** Levels of pmTOR were quantified with each β-actin and mTOR were quantified with each β-actin respectively, then the ratio of pmTOR/mTOR were as below.”

In the published article, four citations were erroneously omitted from the **Discussion**, section, paragraph 6, and were not included in the **References** section. The corrected paragraph and citation information appear below:

“In Figure 4, normalization β-actin is recognized as an internal reference. For most tissues and cells, its expression is abundant and stable. In response to this issue, I think it is necessary to explore the rationality of β-actin as an internal reference. First of all, the actin family includes 6 proteins. The homology between different actins is as high as 90%. There are four muscle tissue-specific β-actin, and the other two are expressed in non-muscle tissues. The sample in this study is brain tissue, not muscle tissue. So specificity is not a problem. According to the following literature reports, there are three cases of β-actin changes. (1) In mouse spinal cord injury or muscle atrophy models, as well as some neurodegenerative diseases, the expression of actin will change (Ferguson et al., 2005; Bauer et al., 2009); (2) Changes in expression levels in different tissues of the same species. Studies have analyzed that the expression levels of actin in muscle, heart, bone, and fat of mice are different (Eaton et al., 2013); (3) Changes in the expression of actin in different parts of the same tissue. In the sciatic nerve tissue of mice, the expression of actin is higher in the proximal tissue than in the distal tissue. Same as above mentioned, effective linear range of β-actin. An ideal internal control should have a wide linear interval to accommodate samples with different protein expression levels. Eaton et al.'s thermal study found that in brain homogenate, the linearity of β-actin was better when the total protein loading amount was between 10–30 μg, and the band signal above 30 μg tended to be saturated. However, the experiment (Dittmer and Dittmer, 2006) found that 2 μg was saturated. This difference may be related to the experimental sample and the sensitivity of fluorescence chromatography. The specific brain samples were collected to investigate the proteins expressions with β-actin as internal control is reasonable and admitted. In fact, there is none even possible minor element to have effect on the expression of β-actin in this study. Of course, we know that the sensitivity of fluorescence chromatography is much higher than that of ECL luminescence method. Based on the above research on β-actin as internal reference combined with the western blot results in Figure 4 of our article, we unanimously agree and recognize that the quantitative method of western blot mentioned by experts is the most correct and accurate, but in our experiment, it did not involve factors that cause actin to change. Any factor, therefore, we adopted an approach also adopted by many other colleagues to compare target proteins. This is of course not as accurate as the quantitative method mentioned by experts, but our method simplifies the steps. Although the process is not precise, it does not affect the final result. Biomedicine is developing rapidly, and western blot, as a protein semi-quantitative technology, is also undergoing innovation.”

The authors apologize for these errors and state that they do not change the scientific conclusions of the article in any way. The original article has been updated.
